# Association Between the Serum Creatinine to Cystatin C Ratio, Physical Activity, and Frailty in Middle-Aged and Older Adults in China: A Nationwide Cohort Study

**DOI:** 10.3390/life15121832

**Published:** 2025-11-28

**Authors:** Kai Song, Chuanwen Yu, Yanwei You

**Affiliations:** 1 School of Physical Education, Qilu Normal University, Jinan 250200, China; songkai@qlnu.edu.cn; 2 School of Physical Education and Health, Heze University, Heze 274015, China; yuchuanwen@hezeu.edu.cn; 3 Division of Sports Science & Physical Education, Tsinghua University, Beijing 100084, China; 4 Saw Swee Hock School of Public Health, National University of Singapore, Singapore 117549, Singapore

**Keywords:** frailty, sarcopenia index, creatinine-to-cystatin C ratio, physical activity, ageing, cohort study

## Abstract

Background: Frailty is a major barrier to healthy ageing, yet early identification strategies remain limited. The serum creatinine-to-cystatin C ratio (sarcopenia index, SI) has emerged as a cost-effective biomarker of muscle mass and function, while physical activity (PA) is a key protective factor. However, their combined role in predicting frailty is unclear. This study aimed to investigate the independent and joint associations of SI and PA with incident frailty in middle-aged and older adults. Methods: We analyzed 5307 participants aged ≥45 years from the China Health and Retirement Longitudinal Study (CHARLS, 2011–2018). SI was calculated from serum creatinine and cystatin C levels, and PA was assessed using standardized questionnaires. Frailty was defined using a 32-item Frailty Index (FI ≥ 0.25). Cox proportional hazards models estimated hazard ratios (HRs) and 95% confidence intervals (CIs) for the associations of SI and PA with incident frailty, adjusting for sociodemographic and health-related factors. Effect modification by PA was formally tested. Results: Over the follow-up period, 1483 participants developed frailty (27.9%). Higher SI was inversely associated with frailty in a dose–response manner: compared with the lowest quartile, HRs (95% CIs) were 0.84 (0.73–0.97) for Q2, 0.83 (0.72–0.96) for Q3, and 0.69 (0.59–0.82) for Q4 (*p*-trend < 0.001). Each 10-unit increase in SI corresponded to a 6% lower frailty risk (HR = 0.94, 95% CI: 0.91–0.97). PA significantly modified this relationship (interaction *p* < 0.05), with the strongest protective effect of SI observed among individuals with low PA, and attenuation at higher PA levels. Conclusions: SI is independently associated with a lower risk of incident frailty, particularly among less physically active individuals. These findings support the potential use of SI as a feasible biomarker for early frailty risk stratification and highlight the importance of integrating biomarker-based screening with lifestyle interventions to prevent frailty.

## 1. Introduction

Frailty is a major global public health challenge, affecting an estimated 12–24% of community-dwelling older adults worldwide, with prevalence increasing sharply with age [1]. In China, recent national surveys indicate that approximately 7–10% of adults aged ≥60 years and up to 20% of those aged ≥80 years are frail, reflecting a rapidly escalating burden amid accelerated population ageing [2,3,4]. Frailty is characterized by progressive declines in physiological reserve and multisystem function, resulting in heightened vulnerability to stressors. Older adults with frailty face increased risks of functional dependence, falls, hospitalization, long-term care needs, and premature mortality, thereby posing a substantial barrier to achieving healthy ageing [5]. At the societal level, frailty imposes considerable clinical and socioeconomic burdens on families, healthcare systems, and public resources. Despite growing recognition of its importance, strategies for early identification and effective prevention remain limited, underscoring the need for accessible biomarkers and modifiable determinants to support timely risk stratification.

The serum creatinine-to-cystatin C ratio, also known as the sarcopenia index (SI), has recently emerged as a promising biomarker for assessing muscle mass and function [6,7]. Unlike traditional diagnostic techniques such as dual-energy X-ray absorptiometry and computed tomography—which are accurate but costly, time-consuming, and unsuitable for large-scale community screening—SI provides a convenient, inexpensive, and radiation-free alternative applicable to both research and clinical practice [8]. Since its introduction by Kim et al., SI has been shown to correlate strongly with muscle mass measured by dual-energy X-ray absorptiometry in ambulatory adults [9] and with muscle quantity quantified by abdominal computed tomography in critically ill patients. Moreover, higher SI has been associated with shorter hospital stays, lower mortality risk among intensive care populations, and reduced adverse cardiovascular events in individuals with coronary artery disease [10,11,12]. These findings highlight SI as a clinically relevant biomarker of muscle-related health outcomes. However, despite accumulating evidence across hospital-based settings, the association between SI and frailty in community-dwelling middle-aged and older adults remains insufficiently examined.

PA is a key modifiable determinant of healthy ageing and one of the most consistently reported protective factors against frailty [13]. Extensive epidemiological evidence demonstrates that regular PA improves muscle strength, enhances physical performance, and reduces the onset and progression of frailty [14,15]. Findings from the English Longitudinal Study of Ageing and prospective cohort studies in Japan indicate that higher levels of PA are associated with substantially lower risks of incident frailty and disability [16,17]. Intervention studies and meta-analyses further show that structured PA programs can reverse or attenuate frailty, reinforcing their central role in preventing age-related functional decline [18]. Nevertheless, although the protective effects of PA are well established, its potential interaction with novel biomarkers such as SI remains poorly understood in large, community-based populations.

Despite increasing recognition of frailty as a major public health concern and emerging evidence regarding the predictive value of SI and the protective role of PA, the interrelationship among these factors remains unclear. Prior studies have typically examined SI in relation to muscle mass, cardiovascular outcomes, or mortality, while evidence on SI and frailty in general populations is scarce. Likewise, although PA consistently reduces frailty risk, it is not known whether PA modifies the association between SI and frailty. Addressing these gaps is essential, as integrating easily obtainable biomarkers with modifiable lifestyle factors may improve early detection and prevention strategies. Therefore, using data from the nationally representative CHARLS, we aimed to investigate the associations of SI and PA with incident frailty among middle-aged and older adults in China, and to further explore potential interactions between SI and PA in predicting frailty risk.

## 2. Methods

### 2.1. Data Sources and Study Design

Our study used longitudinal data from the CHARLS survey from 2011 to 2018. A nationally representative prospective cohort designed to collect extensive socioeconomic and health-related information for aging and health policy research in China http://charls.pku.edu.cn/ (7 July 2025). A total of 17,705 participants were initially recruited from 150 counties/districts across 28 provinces. Baseline interviews were conducted between 2011 and 2012, with follow-up assessments every two years in 2013–2014, 2015–2016, 2017–2018, and 2019–2020. The CHARLS used a probability-proportional-to-size multistage sampling method, including stratification by region and urban–rural classification, as well as clustering at the community or village level. Sampling weights were created to account for different probabilities of selection and to assist with post-stratification adjustments. Analyses were conducted using the nationally weighted CHARLS dataset, which ensures it accurately represents the middle-aged and older Chinese population. Details of the study design have been reported elsewhere [19]. CHARLS was approved by the Institutional Review Board of Peking University (IRB00001052-11015), and all participants provided written informed consent. At each wave, trained interviewers conducted face-to-face surveys using standardized questionnaires to gather information on sociodemographic characteristics, medical history, health behaviors, cognitive function, and depressive symptoms.

To create the analytic cohort, we applied predefined inclusion and exclusion criteria to the baseline sample. Participants were eligible if they: (1) had valid demographic information, including age and sex; (2) were aged 45 years or older at baseline; (3) had available laboratory measurements of serum creatinine and cystatin C for calculating the SI; (4) had complete information on physical activity (PA), as PA was assessed in a randomly selected subsample.

**Figure 1:**
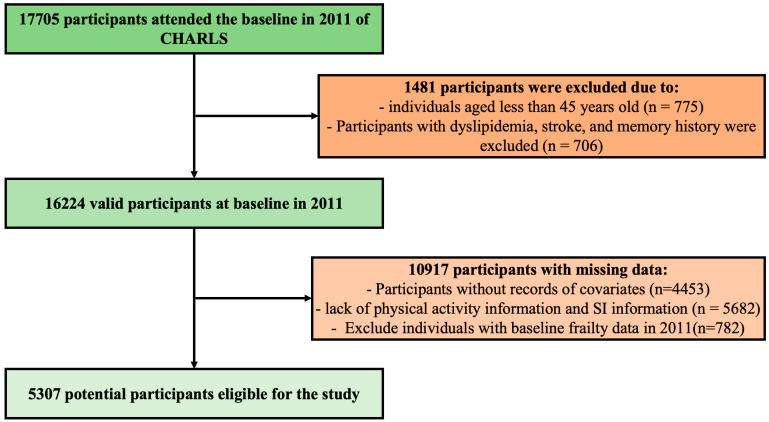
Research design roadmap.

Participants were excluded if they: (1) had missing information on age or sex, or were younger than 45 years at baseline (*n* = 775); (2) lacked PA or social interaction data (*n* = 5682); (3) had a baseline history of dyslipidemia, stroke, or memory-related diseases (*n* = 706) to minimize reverse causation and potential confounding from major health conditions affecting SI or frailty; (4) had missing data on relevant covariates, including sociodemographic and health-related variables (*n* = 4453); (5) were classified as frail at baseline (Frailty Index ≥ 0.25; *n* = 782) to ensure incident frailty was correctly identified during follow-up. After applying these criteria, a total of 5307 participants were included in the final analytic sample. A detailed flowchart of participant selection is provided in Figure 1.

### 2.2. The Serum Creatinine to Cystatin C Ratio

The ratio of serum creatinine to cystatin C, known as the SI, has increasingly been recognized as a surrogate biomarker for muscle mass and function. It is widely acknowledged for its ability to predict and diagnose sarcopenia. Venous blood samples were collected from each participant by trained medical staff at the Chinese Center for Disease Control and Prevention (China CDC) following a standardized protocol after an overnight fast of at least 8 h. Samples were transported at 4 °C to local laboratories for immediate processing and stored at −20 °C. Within two weeks, all specimens were transferred to the China CDC in Beijing and stored at −80 °C until further analysis at the Capital Medical University central laboratory. Serum cystatin C was measured using a particle-enhanced turbidimetric assay, and serum creatinine was determined by the rate-blanked, compensated Jaffe method. The exposure variable in our study was SI from the 2011 CHARLS, which was calculated as:

SI = (Serum creatinine/Serum cystatin C) × 100

Participants were classified into quartiles of SI, resulting in four subgroups for analysis.

### 2.3. Assessment of Frailty

Frailty was assessed using the Frailty Index (FI), derived from data in the CHARLS database [20], to measure the accumulation of age-related health deficits. The FI was created based on 32 health-related variables covering comorbidities, symptoms, functional impairments, and other age-related issues. Each deficit was scored as 0 (absent) or 1 (present), and the FI was calculated as the ratio of deficits present to the total possible (FI = number of deficits/32). The FI score ranged from 0 to 1, with higher scores indicating greater frailty. Consistent with prior research, frailty was defined as FI ≥ 0.25 [21]. To maximize the sample size, individuals with less than 10% missing data across the 32 health-related variables were included. Missing values were not imputed; participants with incomplete data on the components used to calculate the FI were excluded from the analysis. The variables included in the FI construction are listed in Appendix A Table A1.

### 2.4. Assessment of PA

PA was measured using items from the International Physical Activity Questionnaire (IPAQ) included in CHARLS. The questionnaire assessed three intensity-specific activity types: vigorous-intensity activity, moderate-intensity activity, and walking. For each activity type, we first calculated the average daily duration based on questionnaire responses. Categorical responses for duration (<30 min, ≥30 min but <2 h, ≥2 h but <4 h, and ≥4 h per day) were converted into midpoint values (20, 75, 180, and 240 min, respectively). Weekly duration was then obtained by multiplying the average daily duration by the number of days per week reported for each activity type. To account for activity intensity, weekly minutes were converted into metabolic equivalent (MET) minutes per week using standardized IPAQ coefficients: 8.0 METs for vigorous-intensity activity, 4.0 METs for moderate-intensity activity, and 3.3 METs for walking. The total PA score was calculated as the sum of MET-minutes per week across the three activity types. In line with World Health Organization recommendations for middle-aged and older adults, we considered both moderate-intensity and vigorous-intensity activities when classifying overall PA level. According to the IPAQ scoring protocol, participants were subsequently classified into three overall PA levels:

(1) High PA: ≥1500 MET-min/week of vigorous-intensity activity on ≥3 days, or ≥3000 MET-min/week of total PA on ≥7 days;
(2) Moderate PA: ≥600 MET-min/week accumulated on ≥5 days of any combination of walking, moderate-intensity, or vigorous-intensity activity that did not meet the criteria for high PA;
(3) Low PA: those who did not meet the criteria for either moderate or high PA.

Thus, in all analyses, PA level was treated as an ordinal categorical variable with three categories: low, moderate, and high PA, derived from the underlying intensity-specific components (vigorous, moderate, and walking) described above.

### 2.5. Covariates

Baseline sociodemographic, behavioral, and health-related variables were included as covariates in the analyses. Age and sex were obtained from the baseline survey. Educational attainment was categorized as primary school or below, high school, and college or above. Marital status was grouped as married versus never married/separated/widowed. The place of residence was classified as city/town or village. Smoking status was defined as current smoker, ex-smoker, or non-smoker. Alcohol consumption was categorized as drinking less than once per month, drinking more than once per month, and never. Sleep duration was dichotomized as 7–8 h per night (adequate sleep = 1) versus <7 or >8 h (inadequate sleep = 0) [22,23]. Depressive symptoms were assessed using the 10-item Center for Epidemiologic Studies Depression Scale (CES-D-10). A score ≥12 indicated the presence of depressive symptoms [23]. Anthropometric measurements were obtained during the physical examination. Body mass index (BMI) was calculated as weight (kg) divided by height squared (m^2^). Normal weight was defined [24] as 18.5 ≤ BMI < 25 (coded as 1); values outside this range were coded as 0. Waist circumference (WC) was dichotomized [25] as <90 cm for men and <85 cm for women (coded as 1); otherwise coded as 0. Systolic blood pressure (SBP) and diastolic blood pressure (DBP) were measured at baseline and included as continuous variables.

### 2.6. Statistical Analysis

Baseline characteristics of participants were summarized using appropriate descriptive statistics. For continuous variables with a normal distribution, means and standard deviations (SDs) were reported; for non-normally distributed variables, medians and interquartile ranges (IQRs) were presented. Categorical variables were expressed as counts and percentages. Differences in baseline characteristics across groups were examined using analysis of variance (ANOVA) for continuous variables and chi-square tests for categorical variables. Cox proportional hazards regression models were used to estimate the associations of PA levels and SI with the risk of incident frailty, expressed as hazard ratios (HRs) with 95% confidence intervals (CIs). The proportional hazards assumption was checked using Schoenfeld residuals, with no evidence of violation detected (all *p* > 0.05). Multivariable models were adjusted for potential confounders based on prior literature [26], including age, sex, place of residence, marital status, smoking status, alcohol consumption, BMI, WC, sleep duration, depressive symptoms (CES-D category), SBP, and DBP.

To examine whether PA influences the relationship between SI and incident frailty, we conducted stratified analyses based on PA levels (Low, Moderate, High) and evaluated the association between SI and frailty risk within each PA group. To investigate the combined effects of SI and PA on frailty risk, participants were further divided into 12 groups according to SI quartiles (Q1–Q4) and PA levels (Low, Moderate, High). Cox proportional hazards models were employed to calculate HRs and 95% CIs for frailty in each group, with the reference group being participants with the lowest SI (Q1) and the lowest PA level (Low). Effect modification was formally assessed by adding an interaction term between SI (quartiles) and PA levels in the multivariable Cox models.

We performed prespecified subgroup analyses to test the robustness of the link between SI and incident frailty across various participant characteristics. Stratified analyses were conducted based on age (<60 vs. ≥60 years), sex (male vs. female), marital status (married vs. never-married/separated/widowed), residence (rural vs. urban), smoking status (non-smoker, current smoker, ex-smoker), alcohol use (never, less than once a month, more than once a month), and depressive symptoms (CES-D < 12 vs. CES-D ≥ 12). Cox proportional hazards models were applied within each subgroup, adjusting for the same covariates as in the main analyses. The potential effect modification by these factors was further examined by adding multiplicative interaction terms between SI and the stratification variables.

We performed multiple sensitivity analyses to examine the robustness of our findings. First, we excluded participants who developed frailty within the first two years of follow-up to minimize reverse causation. Second, we repeated the analyses after removing those aged 65 or older at baseline. Third, we conducted multiple imputations for covariates, SI, and physical activity (measured in METs) to evaluate the effect of missing data. Multiple imputation was carried out using the “mice ()” function in R (version 4.5.0) with five imputations (m = 5) based on the predictive mean matching (PMM) method and a default predictor matrix. Lastly, we performed E-value analyses. All analyses were conducted using R 4.5.0, and a two-sided *p*-value < 0.05 was considered statistically significant.

## 3. Results

### 3.1. Participant Characteristics

A total of 5307 participants were included in this analysis after excluding individuals based on predefined criteria. The mean age was 59.0 years (SD 9.4), and 48.4% of the participants were male. Table 1 presents the baseline characteristics of participants, stratified by quartiles of the serum creatinine-to-cystatin C ratio (SI). Participants with higher SI levels tended to be younger, male, and better educated. Specifically, the mean age decreased from 62.1 years in Q1 to 56.5 years in Q4 (*p* < 0.001). The proportion of men increased from 26.1% in Q1 to 69.4% in Q4 (*p* < 0.001). The prevalence of college education or higher rose from 1.6% in Q1 to 5.0% in Q4 (*p* < 0.001). For lifestyle factors, the proportions of current smokers and current drinkers increased across SI quartiles (both *p* < 0.001). Conversely, the proportions of non-smokers and never drinkers declined. Sleep duration did not differ significantly across SI groups (*p* = 0.062). There were significant differences in the CES-D category between groups (*p* < 0.001). Higher SI was associated with lower systolic blood pressure (*p* = 0.001) and diastolic blood pressure (*p* < 0.001). In contrast, the distributions of BMI (*p* = 0.254) and WC (*p* = 0.334) did not differ significantly across quartiles.

### 3.2. Association Between Sarcopenia Index and Frailty

**Figure 2:**
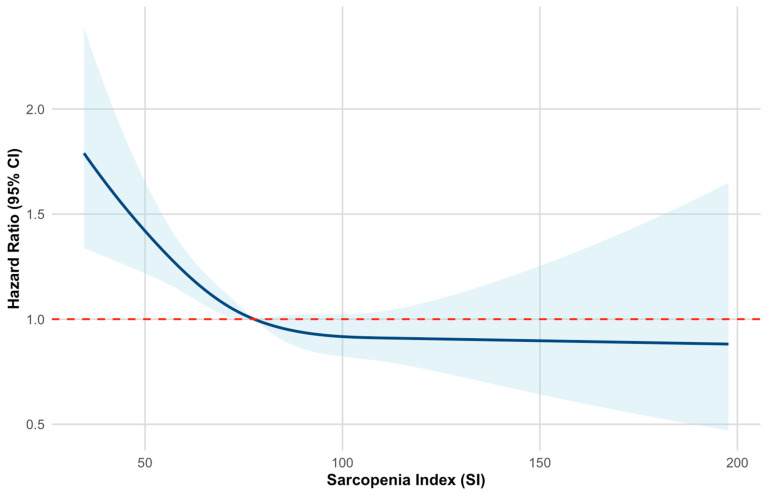
Restricted cubic spline curves for frailty according to the sarcopenia index.

During follow-up, 1483 frailty events occurred among 5307 participants. The overall incidence rate was 27.94%. Table 2 shows that modeling SI as a continuous variable is associated with a lower risk of frailty. In the fully adjusted model (Model 3), a 10-unit increase in SI resulted in a 6% reduction in frailty risk (HR = 0.94, 95% CI: 0.91–0.97, *p* < 0.001). The SI quartiles demonstrated a clear dose–response pattern. Compared to the lowest SI quartile (Q1), higher quartiles were linked to a reduced frailty risk. After full adjustment, the HRs were 0.84 (95% CI: 0.73–0.97) for Q2, 0.83 (95% CI: 0.72–0.96) for Q3, and 0.69 (95% CI: 0.59–0.82) for Q4 (all *p* < 0.05). A significant linear trend was observed across quartiles (*p* for trend < 0.001). Restricted cubic spline (RCS) analysis also confirmed a linear inverse relationship between SI and frailty, with no indication of non-linearity (*p* for non-linearity > 0.05) (Figure 2). Therefore, higher SI consistently predicted a lower risk of frailty across its range. These findings support the associations observed in both quartile and continuous analyses.

### 3.3. The Association of PA Level and Frailty

Following the analysis of the sarcopenia index, we further examined the independent association between PA levels and frailty. As shown in Table 3, a total of 1483 frailty events were documented during the follow-up period. The incidence rates of frailty were 28.2%, 31.4%, and 26.3% in participants with low, moderate, and high PA levels, respectively. In the crude model (Model 1), individuals with moderate PA levels exhibited a significantly higher risk of frailty compared with those with low PA (HR = 1.21, 95% CI: 1.01–1.46, *p* = 0.04). This association remained significant in the fully adjusted model (Model 3: HR = 1.22, 95% CI: 1.01–1.48, *p* = 0.04). Conversely, high PA was not associated with frailty risk in any model (Model 3: HR = 1.03, 95% CI: 0.91–1.17, *p* = 0.60). Trend analyses across PA categories showed no significant linear relationship with frailty risk (all *p* for trend > 0.20).

### 3.4. Examination of Effect Modification by PA

**Figure 3:**
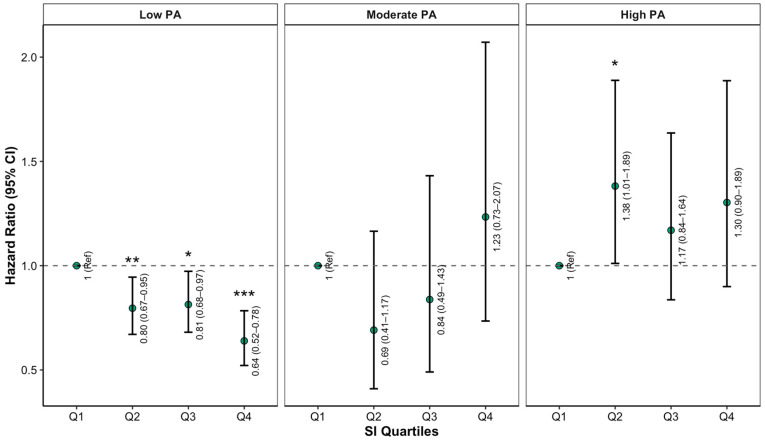
Incidence risk for frailty according to sarcopenia index and PA level categories (* *p* < 0.05, ** *p* < 0.01, *** *p* < 0.001).

We further examined whether PA levels influenced the relationship between the SI and incident frailty (Figure 3). HRs and 95% CIs were derived from Cox proportional hazards models adjusted for age, gender, location, marital status, smoking, drinking, BMI, WC, sleep, CES-D category, SBP, and DBP. Among participants with low PA, higher SI was significantly linked to a lower risk of frailty in a dose–response pattern, with those in the highest quartile of SI (Q4) showing a notably reduced risk compared to Q1 (HR = 0.62, 95% CI: 0.50–0.76). A similar protective trend appeared in the moderate PA group, although the associations were weaker and only borderline significant in some quartiles. Conversely, in individuals with high PA, the link between SI and frailty risk was mostly diminished and no longer statistically significant across SI quartiles. Importantly, interaction analysis confirmed a significant modifying effect of PA on the relationship between SI and frailty (*p* for interaction < 0.05).

### 3.5. Subgroup Analyses and Sensitivity Analyses

**Figure 4:**
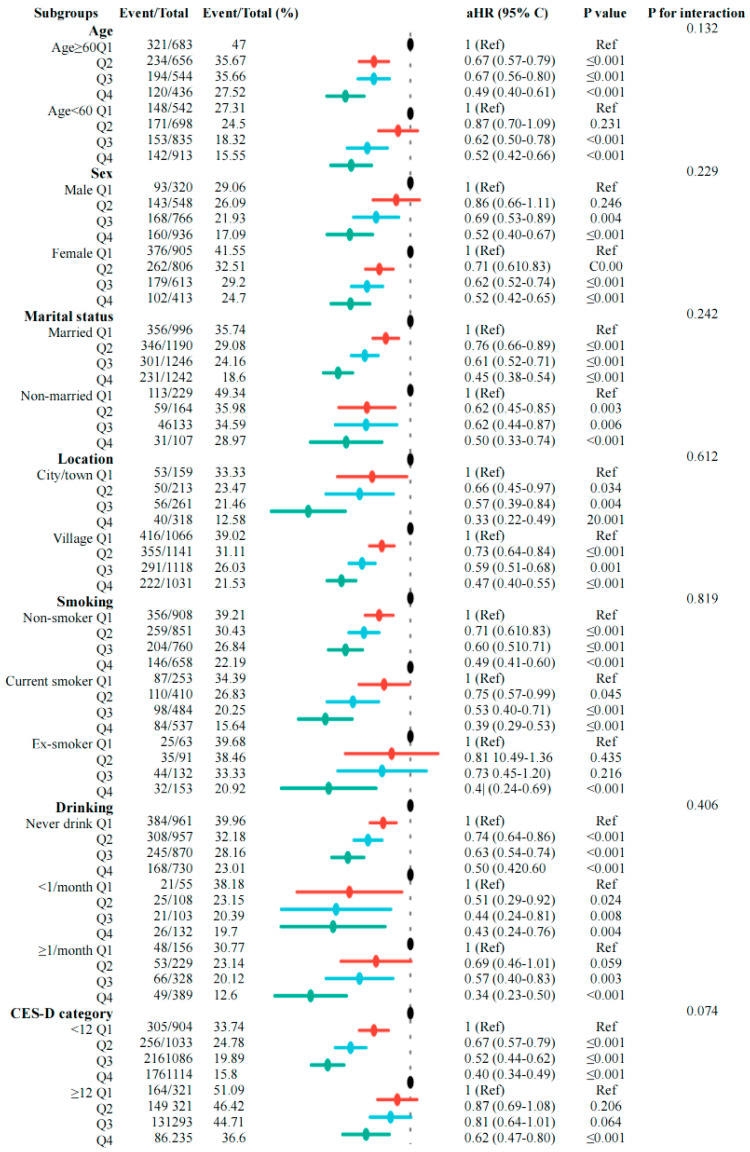
Subgroup and interaction analyses between the sarcopenia index (quartiles 1–4) and risk for frailty across various subgroups in model 3.

To further evaluate the robustness of the link between the sarcopenia index and incident frailty, we performed subgroup analyses based on age, sex, marital status, location, smoking, drinking, and CES-D category (Figure 4). Overall, the associations were consistent across most subgroups, with higher quartiles of the sarcopenia index generally linked to a lower risk of frailty. Although the hazard ratios’ strength varied slightly among different groups, the confidence intervals mostly overlapped, and no statistically significant interactions were found (all *p* values for interaction > 0.05). Notably, the inverse relationship between the sarcopenia index and frailty appeared more evident among older participants, males, or those with higher BMI; however, these differences did not reach statistical significance. Similar patterns appeared across other subgroups, suggesting that the relationship between the sarcopenia index and frailty was broadly consistent and not affected by baseline characteristics. We also performed additional sensitivity analyses to confirm the robustness of our results. The link between the serum creatinine-to-cystatin C ratio and frailty risk stayed mostly the same after (I) excluding participants with a follow-up of two years or less to reduce potential reverse causation (Appendix A Table A2), (II) removing those aged 65 or older at baseline (Appendix A Table A3), (III) using multiple imputation for missing data on covariates, SI, and physical activity (METs) (Appendix A Table A4, Table A5 and Table A6), and (IV) conducting E-value analyses (Appendix A Table A7), which showed that a significant amount of unmeasured confounding would be needed to fully explain the observed association.

## 4. Discussion

This study aimed to investigate the independent and joint associations of the serum creatinine-to-cystatin C ratio (sarcopenia index, SI) and PA with incident frailty in a nationally representative cohort of middle-aged and older Chinese adults. In this longitudinal analysis of 5307 participants, we found that higher SI was consistently associated with a lower risk of developing frailty, demonstrating a clear dose–response pattern. In contrast, PA showed a non-linear relationship with frailty risk, with individuals reporting moderate PA levels exhibiting a slightly higher risk than those with low PA, while high PA was not significantly associated with frailty. Furthermore, a significant interaction between SI and PA indicated that the protective effect of SI on frailty was strongest among individuals with lower PA levels. These findings highlight the potential value of integrating biomarker-based assessment with lifestyle factors to improve early detection and prevention of frailty.

Our results extend previous evidence on SI, which has been widely investigated as a surrogate marker for muscle mass and function. Earlier studies demonstrated that SI correlated strongly with muscle mass measured by dual-energy X-ray absorptiometry and computed tomography, and predicted adverse outcomes such as prolonged hospitalization, intensive care mortality, and cardiovascular events [27]. However, prior work has primarily focused on clinical populations, while evidence on the role of SI in predicting frailty—a multidimensional geriatric syndrome—remains scarce [28]. By documenting this association in a community-based, nationwide cohort, our study provides novel evidence that SI may serve as a practical tool for identifying individuals at increased frailty risk.

The observed interaction between SI and PA provides valuable insights. Low SI likely indicates reduced muscle reserve, which increases the risk of frailty through mechanisms such as chronic inflammation, insulin resistance, impaired energy metabolism, and neuromuscular dysfunction [29]. In contrast, PA has well-known benefits for muscle growth, mitochondrial health, cardiopulmonary fitness, and systemic anti-inflammatory effects [30,31]. Our previous research shows that physical activity influences molecular and biological aging processes. For example, You et al. [32] found that accelerometer-measured PA patterns were linked to phenotypic age, while later studies showed that PA relates to DNA methylation-based epigenetic clocks [33] and telomere length changes [34]. These results suggest that PA impacts aging not only through physical functions but also via cellular and molecular pathways. Building on this emerging evidence, our study offers new insights by combining a common biochemical marker—the serum creatinine-to-cystatin C ratio—with PA to examine their combined effects on frailty. While prior work, such as da Silva et al. [31] has highlighted the connection between low PA and frailty, our findings further reveal that the negative effect of low SI on frailty risk is especially pronounced among those with low PA. This suggests that PA may biologically counteract the adverse effects of reduced muscle reserve, possibly through improved mitochondrial activity, reduced inflammation, and enhanced metabolic balance. Furthermore, the unexpectedly higher frailty risk observed in the moderate PA group may be due to reverse causation, heterogeneity within the IPAQ-defined moderate category, and differences in baseline health status across PA levels. These factors probably weakened the expected dose–response relationship and caused a non-linear pattern.

This study has several strengths. First, it was based on the China Health and Retirement Longitudinal Study, a nationally representative cohort with standardized data collection and follow-up, enhancing generalizability. Second, the use of SI, derived from routine blood tests, provides a simple, low-cost, and radiation-free alternative to traditional sarcopenia assessments. Third, frailty was operationalized using a validated Frailty Index, capturing multisystem deficits rather than relying on a single dimension. Finally, multiple sensitivity analyses confirmed the robustness of the findings.

Several limitations of the present study should be acknowledged. First, the absence of biochemical indicators such as creatinine and cystatin C limits the ability to account for individual differences in kidney and muscle function, which may independently influence exercise-related behaviors. Future research should incorporate these biomarkers to provide a more comprehensive understanding of their physiological and behavioral associations. Second, PA was self-reported through questionnaires, which may introduce recall bias and potential misclassification. Third, SI, although convenient, are influenced by renal function, dietary intake, and inflammatory status; therefore, residual confounding may persist despite statistical adjustments. Moreover, both SI and PA were measured only at baseline, precluding the assessment of temporal variations or causal directionality. Finally, the observational design inherently limits causal inference, and the possibility of reverse causation cannot be fully excluded. Caution is also warranted when generalizing these findings to non-Chinese populations, given potential cultural and biological differences.

Despite these limitations, our findings have important implications for clinical practice and public health. SI, as a routinely available biomarker, could be incorporated into community health screening to identify individuals at higher frailty risks, particularly in resource-limited settings. In addition, the modifying role of PA emphasizes the importance of lifestyle interventions in mitigating the adverse effects of low muscle reserve. Taken together, these results highlight the need for integrated strategies that combine biomarker-based screening with promotion of physical activity to prevent or delay frailty. Future research should validate our findings in independent cohorts, examine optimal SI cut-off values for risk prediction, and incorporate repeated measurements of SI and PA to assess dynamic changes. Moreover, interventional studies are warranted to test whether improving SI through nutritional and exercise interventions, particularly resistance training, can effectively reduce frailty incidence and progression.

## 5. Conclusions

In this nationwide cohort of middle-aged and older Chinese adults, we found that a higher serum creatinine-to-cystatin C ratio was significantly associated with a reduced risk of incident frailty. Importantly, PA modified this relationship, with the protective effect of SI being most evident among individuals with low PA levels. These findings suggest that SI, as an easily obtainable and cost-effective biomarker, may serve as a valuable tool for early frailty risk stratification in community settings. Moreover, the observed interaction highlights the importance of integrating biomarker-based assessments with lifestyle interventions to more effectively prevent or delay frailty. Future studies are needed to validate these findings in diverse populations, to establish optimal SI cut-off values for clinical practice, and to evaluate whether targeted strategies combining nutritional and exercise interventions can improve SI and reduce frailty risk.

## Figures and Tables

**Table 1:** Baseline characteristics of participants stratified by quartiles of the serum creatinine-to-cystatin C ratio.

Variables	Total (n = 5307)	Quartiles of SI				*p* Value
		Q1 (n = 1225)	Q2 (n = 1354)	Q3 (n = 1379)	Q4 (n = 1379)	
Age (mean (SD))	59.0 (9.4)	62.1 (10.0)	60.0 (9.3)	57.9 (9.0)	56.5 (8.2)	<0.001
Sex (%)						<0.001
Female	2737 (51.6)	905 (73.9)	806 (59.5)	613 (44.5)	413(30.6)	
Male	2570 (48.4)	320 (26.1)	548 (40.5)	766 (55.5)	936(69.4)	
Education (%)						<0.001
College or above	179 (3.4)	20 (1.6)	40 (3.0)	51 (3.7)	68 (5.0)	
High school	1461 (27.5)	215 (17.6)	325 (24.0)	426 (30.9)	495(36.7)	
Primary school or below	3667 (69.1)	990 (80.8)	989 (73.0)	902 (65.4)	786(58.3)	
Location (%)						<0.001
City/town	951 (17.9)	159 (13.0)	213 (15.7)	261 (18.9)	318(23.6)	
Village	4356 (82.1)	1066 (87.0)	1141 (84.3)	1118 (81.1)	1031(76.4)	
Marital status (%)						<0.001
Marital	4674 (88.1)	996 (81.3)	1190 (87.9)	1246 (90.4)	1242(92.1)	
never-married /separated/widowed	633 (11.9)	229 (18.7)	164 (12.1)	133 (9.6)	107 (7.9)	
Smoking (%)						<0.001
Current smoker	1684 (31.8)	253 (20.7)	410 (30.3)	484 (35.2)	537(39.8)	
Ex-smoker	439 (8.3)	63 (5.1)	91 (6.7)	132 (9.6)	153(11.4)	
Non-smoker	3177 (59.9)	908 (74.2)	851 (62.9)	760 (55.2)	658(48.8)	
Drinking (%)						<0.001
Drinks but less than once a month	398 (7.9)	55 (4.7)	108 (8.3)	103 (7.9)	132(10.6)	
Drink more than once a month	1102 (22.0)	156 (13.3)	229 (17.7)	328 (25.2)	389(31.1)	
never	3518 (70.1)	961 (82.0)	957 (74.0)	870 (66.9)	730(58.4)	
Sleep (%)						0.062
0	3051 (57.5)	743 (60.7)	779 (57.5)	773 (56.1)	756(56.0)	
1	2256 (42.5)	482 (39.3)	575 (42.5)	606 (43.9)	593(44.0)	
SBP (mean (SD))	130.0 (21.3)	132.0 (22.4)	130.3 (22.3)	129.1 (20.9)	128.8 (19.5)	0.001
DBP (mean (SD))	75.3 (12.0)	74.7 (11.9)	74.4 (11.9)	75.6 (12.1)	76.4 (11.9)	<0.001
CES-D category (%)						<0.001
<12	4137 (78.0)	904 (73.8)	1033 (76.3)	1086 (78.8)	1114(82.6)	
≥12	1170 (22.0)	321 (26.2)	321 (23.7)	293 (21.2)	235(17.4)	
BMI (%)						0.254
0	1912 (36.0)	461 (37.6)	462 (34.1)	491 (35.6)	498(36.9)	
1	3395 (64.0)	764 (62.4)	892 (65.9)	888 (64.4)	851(63.1)	
WC (%)						0.334
0	2092 (39.4)	504 (41.1)	521 (38.5)	525 (38.1)	542(40.2)	
1	3215 (60.6)	721 (58.9)	833 (61.5)	854 (61.9)	807(59.8)	

Data are the mean (SD), median [IQR] or number (%), as appropriate. BMI indicates body mass index; WC waist circumference; CES-D; Center for Epidemiologic Studies Depression Scale; DBP, diastolic blood pressure; SBP, systolic blood pressure; and SI, sarcopenia index.

**Table 2:** Association between the sarcopenia index and frailty.

Categories	Events, n	Total, n	Incidence Rate	Model 1		Model 2		Model 3	
				HR (95% CI)	*p* Value	HR (95% CI)	*p* Value	HR (95% CI)	*p* Value
frailty									
Continuous	1483	5307	27.94%	0.94 (0.88–1.00)	0.06	0.94 (0.91–0.97)	<0.001	0.94 (0.91–0.97)	<0.001
Quartiles									
Q1	469	1225	38.29%	Ref		Ref		Ref	
Q2	405	1354	29.91%	0.72 (0.63–0.82)	<0.001	0.82 (0.72–0.95)	0.006	0.84 (0.73–0.97)	0.01
Q3	347	1379	25.16%	0.58 (0.51–0.67)	<0.001	0.83 (0.71–0.96)	0.013	0.83 (0.72–0.96)	0.01
Q4	262	1349	19.42%	0.43 (0.37–0.51)	<0.001	0.70 (0.59–0.82)	<0.001	0.69 (0.59–0.82)	<0.001
*p* for trend					<0.001		<0.001		<0.001

CI, confidence interval; HR, hazard ratio; Ref, reference; Q, Quantiles. Model 1: Crude model; Model 2: Adjusted for age, gender, location, marital status, smoking, drinking; Model 3: Further adjusted for BMI, WC, Sleep, CES-D category, SBP, DBP. *p*-value for linear trend calculated from category median values. Continuous intakes were calculated by 10 10-unit increase.

**Table 3:** Association between the PA level and frailty.

Categories	Events, n	Total, n	Incidence Rate	Model 1		Model 2		Model 3	
				HR (95% CI)	*p* Value	HR (95% CI)	*p* Value	HR (95% CI)	*p* Value
PA level									
Low	971	3439	28.23%	Ref		Ref		Ref	
Moderate	125	398	31.41%	1.21 (1.01–1.46)	0.04	1.21 (0.99–1.46)	0.05	1.22 (1.01–1.48)	0.04
High	387	1470	26.33%	0.91 (0.81–1.02)	0.12	1.04 (0.92–1.17)	0.57	1.03 (0.91–1.17)	0.60
*p* for trend					0.20		0.41		0.42

CI, confidence interval; HR, hazard ratio; Ref, reference; Q, Quantiles. Model 1: Crude model; Model 2: Adjusted for age, gender, location, marital status, smoking, drinking; Model 3: Further adjusted for BMI, WC, Sleep, CES-D category, SBP, DBP. *p*-value for linear trend calculated from category median values.

## Data Availability

The CHARLS dataset is freely available to all researchers in related fields on request. Researchers can gain access to the data http://charls.pku.edu.cn/ (7 July 2025). And the datasets used and/or analyzed in this current study are available from the corresponding author on reasonable request.

## Abbreviations

The following abbreviations are used in this manuscript:

SI	sarcopenia index
PA	Physical activity
CHARLS	China Health and Retirement Longitudinal Study
FI	Frailty Index

## Appendix A

**Table A1:** 32 items to construct the frailty index.

No	Description of the Items	Cut-off Value
1	Self-reported diagnosis of hypertension by a doctor	Yes = 1, No = 0
2	Self-reported diagnosis of diabetes by a doctor	
3	Self-reported diagnosis of heart attack, coronary heart disease, angina, congestive heart failure, or other heart problems by a doctor	
4	Self-reported diagnosis of stroke by a doctor	
5	Self-reported diagnosis of cancer by a doctor	
6	Self-reported diagnosis of arthritis by a doctor	
7	Self-reported diagnosis of chronic lung diseases by a doctor	
8	Self-reported diagnosis of asthma by a doctor	
9	Self-reported diagnosis of emotional, nervous, or psychiatric problems by a doctor	
10	Self-reported diagnosis of memory-related disease by a doctor	
11	Self-reported vision problems	
12	Self-reported hearing problems	
13	Difficulty with dressing	Did not have any problems with the activity = 0; some difficulty with the activity or could not do the activity = 1.
14	Difficulty with bathing or showering	
15	Difficulty with eating	
16	Difficulty with getting in and out of bed	
17	Difficulty with using the toilet	
18	Difficulty with managing money	
19	Difficulty with taking medications	
20	Difficulty with shopping for groceries	
21	Difficulty with preparing meals	
22	Difficulty with doing housework	
23	Difficulty with walking 100 yards	
24	Difficulty with getting up from a chair after sitting for long periods	
25	Difficulty with climbing several flights of stairs without resting	
26	Difficulty with lifting or carrying weights over 10 pounds/jins	
27	Difficulty with picking up a coin from the table	
28	Difficulty with stooping, kneeling, or crouching	
29	Difficulty with reaching arms above shoulder level	
30	Self-reported health	Poor/fair = 1, excellent/very good/or good = 0
31	Depressive symptoms: CESD-10 questionnaire	CESD-10 > 10 = 1, ≤10 = 0
32	Cognition: (memory test score + orientation test score)/14	Continuous, ranging from 0 to 1

**Table A2:** Hazard ratios and 95% CI of the sarcopenia index for risk of frailty among participants with a duration of follow-up of more than two years.

Categories	Events, n	Total, n	Incidence Rate	Model 1		Model 2		Model 3	
				HR (95% CI)	*p* Value	HR (95% CI)	*p* Value	HR (95% CI)	*p* Value
frailty									
Continuous	850	3849	22.84%	0.91(0.89–1.00)	0.05	0.92(0.98–0.99)	<0.001	0.93(0.94–0.99)	<0.001
Quartiles									
Q1	224	715	31.33%	Ref		Ref		Ref	
Q2	240	911	26.34%	0.85(0.74–1.05)	<0.1	0.89(0.0.74–1.05)	0.198	0.93(0.79–1.15)	0.01
Q3	272	1078	25.23%	0.67(0.55–0.79)	<0.001	0.81(0.67–0.98)	0.024	0.85(0.68–1.03)	0.01
Q4	114	1145	9.96%	0.55(0.44–0.65)	<0.001	0.72(0.59–0.88)	<0.001	0.74(0.57–0.91)	<0.001
*p* for trend					<0.001		<0.001		<0.001

CI, confidence interval; HR, hazard ratio; Ref, reference; Q, Quantiles. Model 1: Crude model; Model 2: Adjusted for age, gender, location, marital status, smoking, drinking; Model 3: Further adjusted for BMI, WC, Sleep, CES-D category, SBP, DBP. *p*-value for linear trend calculated from category median values. Continuous intakes were calculated by 10 10-unit increase.

**Table A3:** Hazard ratios and 95% CI of the sarcopenia index for risk of frailty among participants aged 65 years or younger.

Categories	Events, n	Total, n	Incidence Rate	Model 1		Model 2		Model 3	
				HR (95% CI)	*p* Value	HR (95% CI)	*p* Value	HR (95% CI)	*p* Value
frailty									
Continuous	878	3839	22.87%	0.95(0.87–1.00)	0.05	0.91(0.97–0.99)	<0.001	0.92(0.95–0.98)	<0.001
Quartiles									
Q1	219	720	30.42%	Ref		Ref		Ref	
Q2	244	924	26.41%	0.84(0.70–1.01)	<0.1	0.88(0.0.73–1.07)	0.198	0.92(0.77–1.11)	0.01
Q3	221	1063	20.79%	0.63(0.53–0.77)	<0.001	0.80(0.66–0.97)	0.024	0.84(0.69–1.01)	0.01
Q4	194	1132	17.14%	0.51(0.42–0.62)	<0.001	0.70(0.57–0.87)	<0.001	0.73(0.59–0.90)	<0.001
*p* for trend					<0.001		<0.001		<0.001

CI, confidence interval; HR, hazard ratio; Ref, reference; Q, Quantiles. Model 1: Crude model; Model 2: Adjusted for age, gender, location, marital status, smoking, drinking; Model 3: Further adjusted for BMI, WC, Sleep, CES-D category, SBP, DBP. *p*-value for linear trend calculated from category median values. Continuous intakes were calculated by 10 10-unit increase.

**Table A4:** Baseline Characteristics of All Participants Stratified by Quartiles of Serum Creatinine to Cystatin C Ratio (imputation result).

Variables	Total (n = 15696)	Quartiles of SI				*p* Value
		Q1(n = 2009)	Q2(n = 1993)	Q3(n = 1986)	Q4(n = 1971)	
**Age (mean (SD))**	59.0 (9.8)	64.0 (10.6)	61.1 (9.8)	58.7 (9.2)	56.8 (8.7)	<0.001
**Sex (%)**						<0.001
Female	7994 (50.9)	1475 (73.4)	1183 (59.4)	889 (44.7)	606 (30.7)	
Male	7702 (49.1)	534 (26.6)	810 (40.6)	1100 (55.3)	1365 (69.3)	
**Education (%)**						<0.001
College or above	708 (4.5)	25 (1.2)	66 (3.3)	82 (4.1)	118 (6.0)	
High school	4534 (28.9)	322 (16.0)	439 (22.0)	571 (28.7)	700 (35.5)	
Primary school or below	10,451 (66.6)	1662 (82.7)	1488 (74.7)	1336 (67.2)	1153 (58.5)	
**Location (%)**						<0.001
City/town	3457 (22.0)	264 (13.1)	347 (17.4)	397 (20.0)	487 (24.7)	
Village	12,227 (78.0)	1744 (86.9)	1645 (82.6)	1590 (80.0)	1484 (75.3)	
**Marital status (%)**						<0.001
Marital	13,688 (87.2)	1574 (78.3)	1706 (85.6)	1781 (89.5)	1800 (91.3)	
never-married /separated/widowed	2006 (12.8)	435 (21.7)	287 (14.4)	208 (10.5)	171 (8.7)	
**Smoking (%)**						<0.001
Current smoker	4511 (29.9)	412 (20.8)	562 (28.7)	667 (34.4)	706 (36.9)	
Ex-smoker	1264 (8.4)	116 (5.9)	136 (6.9)	189 (9.7)	237 (12.4)	
Non-smoker	9319 (61.7)	1451 (73.3)	1259 (64.3)	1084 (55.9)	970 (50.7)	
**Drinking (%)**						<0.001
Drinks but less than once a month	1248 (8.6)	94 (4.9)	147 (7.8)	149 (8.0)	187 (10.4)	
Drink more than once a month	2930 (20.1)	241 (12.7)	311 (16.5)	439 (23.5)	532 (29.6)	
never	10,383 (71.3)	1566 (82.4)	1422 (75.6)	1277 (68.5)	1077 (60.0)	
**Sleep (%)**						0.002
0	8493 (58.9)	1214 (63.4)	1137 (59.7)	1112 (59.0)	1076 (57.5)	
1	5929 (41.1)	701 (36.6)	767 (40.3)	774 (41.0)	794 (42.5)	
SBP (mean (SD))	130.5 (21.5)	133.5 (22.9)	131.3 (22.5)	130.2 (21.7)	129.7 (20.1)	<0.001
DBP (mean (SD))	75.8 (12.1)	74.9 (11.9)	74.7 (12.1)	75.9 (12.4)	76.5 (11.9)	<0.001
CES-D category (%)						<0.001
<12	10411 (72.4)	1251 (65.2)	1319 (69.2)	1366 (72.7)	1435 (77.2)	
≥12	3963 (27.6)	668 (34.8)	588 (30.8)	514 (27.3)	424 (22.8)	
BMI (%)						0.446
0	4457 (36.4)	652 (38.2)	612 (35.7)	607 (36.1)	596 (37.2)	
1	7779 (63.6)	1057 (61.8)	1102 (64.3)	1074 (63.9)	1007 (62.8)	
WC (%)						0.067
0	8250 (52.6)	1002 (49.9)	936 (47.0)	960 (48.3)	1003 (50.9)	
1	7446 (47.4)	1007 (50.1)	1057 (53.0)	1029 (51.7)	968 (49.1)	

Data are the mean (SD), median [IQR] or number (%), as appropriate. BMI indicates body mass index; DBP, diastolic blood pressure; SBP, systolic blood pressure; and SI, sarcopenia index.

**Table A5:** Association between the sarcopenia index and frailty (imputation result).

Categories	Events, n	Total, n	Incidence Rate	Model 1		Model 2		Model 3	
				HR (95% CI)	*p* Value	HR (95% CI)	*p* Value	HR (95% CI)	*p* Value
frailty									
Continuous	2890	7959	18.4	26.9	0.95 (0.9–0.98)	0.004	0.94 (0.9–0.96)	<0.001	0.93 (0.9–0.96)
Quartiles									
Q1	930	2009	46.3	38	Ref	—	Ref	—	Ref
Q2	760	1993	38.1	27.1	0.74 (0.6–0.83)	<0.001	0.82 (0.7–0.93)	0.002	0.83 (0.7–0.94)
Q3	640	1986	32.2	23.1	0.59 (0.52–0.68)	<0.001	0.77 (0.6–0.89)	<0.001	0.78 (0.6–0.89)
Q4	560	1971	57.7	20.6	0.47 (0.4–0.55)	<0.001	0.71 (0.6–0.83)	<0.001	0.69 (0.5–0.81)
*p* for trend					<0.001		<0.001		<0.001

CI, confidence interval; HR, hazard ratio; Ref, reference; Q, Quantiles. Model 1: Crude model; Model 2: Adjusted for age, gender, location, marital status, smoking, drinking; Model 3: Further adjusted for BMI, WC, Sleep, CES-D category, SBP, DBP. *p*-value for linear trend calculated from category median values. Continuous intakes were calculated by 10 10-unit increase.

**Table A6:** Association between the PA level and frailty (imputation result).

Categories	Events, n	Total, n	Incidence Rate	Model 1		Model 2		Model 3	
				HR (95% CI)	*p* Value	HR (95% CI)	*p* Value	HR (95% CI)	*p* Value
PA level									
Low	1820	6350	28.7	Ref	—	Ref	—	Ref	—
Moderate	370	1225	30.2	1.10 (0.98–1.24)	0.11	1.08 (0.96–1.22)	0.18	1.07 (0.95–1.21)	0.22
High	640	2650	24.2	0.88 (0.80–0.97)	0.011	0.86 (0.78–0.95)	0.004	0.85 (0.77–0.94)	0.002
*p* for trend				0.02		0.008		0.005	

CI, confidence interval; HR, hazard ratio; Ref, reference; Q, Quantiles. Model 1: Crude model; Model 2: Adjusted for age, gender, location, marital status, smoking, drinking; Model 3: Further adjusted for BMI, WC, Sleep, CES-D category, SBP, DBP. *p*-value for linear trend calculated from category median values.

**Table A7:** Sensitivity analyses: E-values for the association between sarcopenia index and risk of frailty.

Variables	E-values * for HR (and for CI)
sarcopenia index	2.20 (2.75)

CI, confidence interval; HR, odd ratio. * E-value represents the minimum strength of association needed between an unmeasured confounder and both the exposure and the outcome to fully explain away the exposure-outcome association.
